# Symptomatic abdominal aortic aneurysm in a patient with a horseshoe kidney

**DOI:** 10.1590/1677-5449.200088

**Published:** 2020-12-11

**Authors:** Geciana Maria Araujo Coelho, Ranielli Auxiliadora Assem França, Renan Danilo Lima da Rocha, Mariana de Oliveira Pantoja, Patricia de Souza Lacerda, Jose Emerson dos Santos Souza, Marcos Velludo Bernardes, Leonardo Pessoa Cavalcante

**Affiliations:** 1 Universidade Federal do Amazonas, Hospital Universitário Francisca Mendes, Serviço de Cirurgia Vascular e Endovascular, Manaus, AM, Brasil.; 2 Universidade Federal do Amazonas – UFAM, Hospital Universitário Getúlio Vargas, Serviço de Cirurgia Vascular, Manaus, AM, Brasil.; 3 Fundação Hospital Adriano Jorge – HUGV, Serviço de Cirurgia Geral, Manaus, AM, Brasil.; 4 Universidade do Estado do Amazonas – UEA, Escola Superior de Ciências da Saúde, Departamento de Clínica Cirúrgica, Manaus, AM, Brasil.; 5 Universidade Federal do Amazonas – UFAM, Faculdade de Medicina, Programa de Pós-graduação em Cirurgia - PPGRACI, Manaus, AM, Brasil.; 6 Universidade Federal do Amazonas – UFAM, Faculdade de Medicina, Departamento de Clínica Cirúrgica, Manaus, AM, Brasil.

**Keywords:** congenital abnormalities, aortic aneurysm, abdominal aortic aneurysm, fused kidney, kidney/abnormalities

## Abstract

Horseshoe kidney is the most common congenital renal anomaly, occurring in 0.15-0.25% of newborns. The association of a horseshoe kidney with an abdominal aortic aneurysm is rare. Only 0.12% of patients requiring abdominal aortic repair have a horseshoe kidney. This therapeutic challenge constitutes a patient presenting with a symptomatic abdominal aortic aneurysm and a horseshoe kidney. The horseshoe kidney was supplied by 4 renal arteries, 2 of which emerged from the aneurysmal sac. The patient underwent urgent open repair, with transperitoneal exposure, interposition of a bifurcated aorto-bi-iliac Dacron graft and re-implantation of the 2 anomalous renal arteries on the Dacron main body. Postoperatively, the patient was discharged from the intensive care unit on day 3, and discharged home on day 8, maintaining normal serum creatinine.

## INTRODUCTION

Horseshoe kidney is the most common congenital renal anomaly, occurring in 0.15 to 0.25% of newborns, with a predominance in males at a ratio of 2:1.^1^ The association between horseshoe kidney and abdominal aortic aneurysm (AAA) is rare, being present in only 0.12% of patients who undergo surgical, open or endovascular, AAA repair.^2^ Treatment of AAA in a patient with a horseshoe kidney involves certain peculiarities: 1) the renal parenchyma covering the AAA; 2) anomalies of renal arterial supply; and 3) anomalies related to the urinary collector system.^3^

## PART I: CLINICAL SITUATION

A 65-year-old male smoker with an asymptomatic AAA with a diameter of 7.2 cm was being monitored in an outpatient setting, and undergoing preoperative exams for surgical risk stratification. During outpatient follow-up, he presented with an intense acute abdominal pain, irradiating bilaterally to the lumbar region, without hypotension, and was urgently admitted to the hospital.

**Figure 1:**
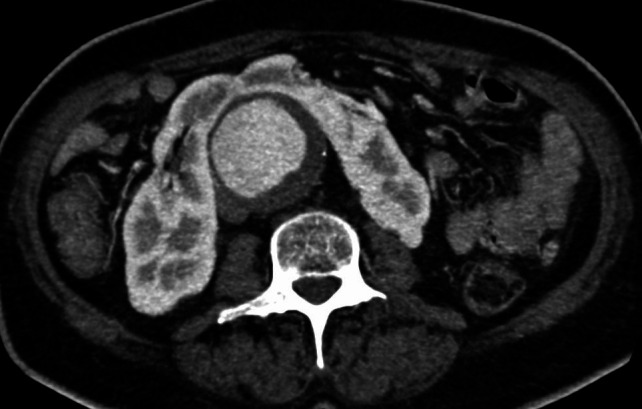
Horseshoe kidney with fusion anterior to the abdominal aortic aneurysm.

**Figure 2:**
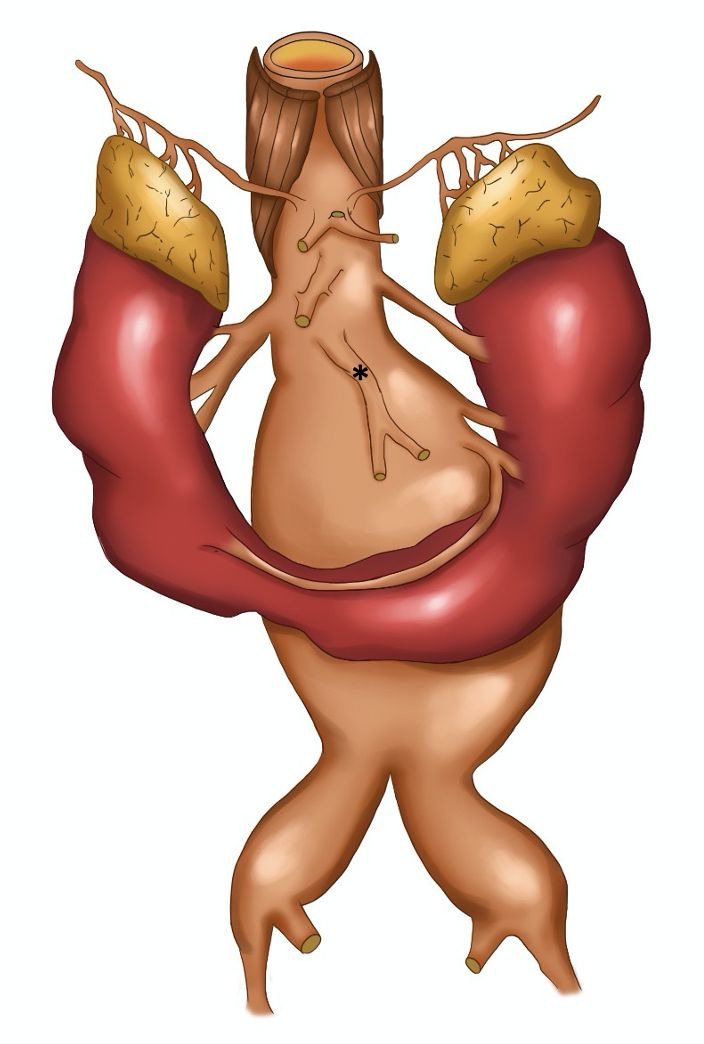
Schematic drawing illustrating arterial supply to the horseshoe kidney and the origins of the digestive arteries. *: Inferior mesenteric artery with high origin.

Preoperative angiotomography revealed a horseshoe kidney, fused at the inferior renal poles, with a narrow fibrotic component at the fusion zone, anterior to the AAA (Figure 1). The majority of the supply to the right kidney was provided by a main renal artery, emerging a few millimeters below the superior mesenteric artery; the left kidney was supplied by a left superior polar artery which also emerged a few millimeters below the superior mesenteric artery and by a main (hilar) renal artery, emerging from the proximal segment of the AAA and measuring 3.5 mm in diameter. The inferior renal poles were supplied by a second artery (accessory), which also originated from the proximal segment of the AAA, approximately 3 mm below the main left renal artery, and also measuring 3.5 mm in diameter. The accessory left renal artery bifurcated into two branches: one supplying the left inferior renal pole and the other, which crossed over anterior to the AAA, just above the isthmus of the horseshoe kidney, supplied the right inferior renal pole (Figure 2). The inferior mesenteric artery had a high origin, from the distal segment of the proximal neck of the AAA, measured 6 mm in diameter, and was patent. The AAA began 25 mm below the right renal artery, with a 60 degrees anterior proximal neck angulation, and involved both common iliac arteries (29 mm dilatation on the right side and 39 mm dilatation on the left side).

In this situation, there were a number of treatment possibilities: 1) endovascular treatment; 2) open surgical treatment with a transperitoneal approach; or 3) open surgical treatment with a retroperitoneal approach. The patient gave consent for publication of his case.

## PART II: WHAT WAS DONE

**Figure 3:**
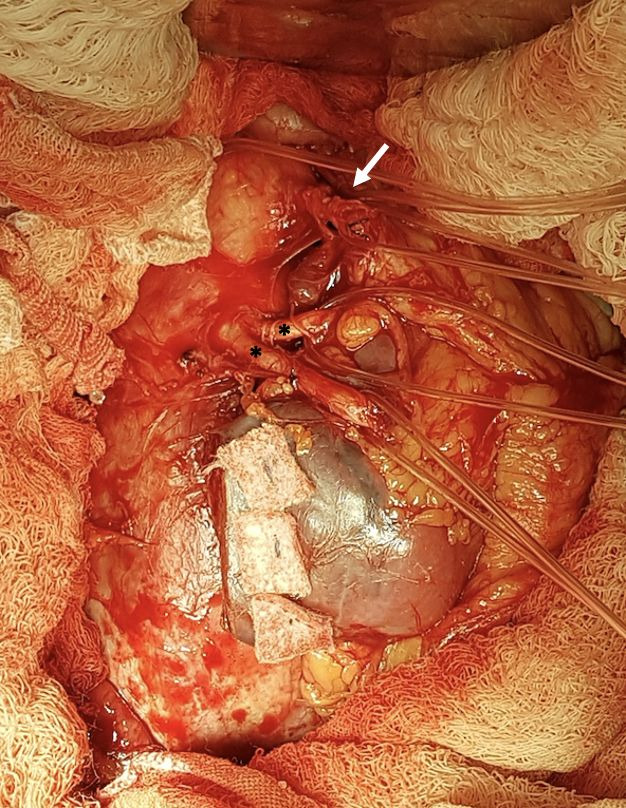
Surgical exposure of the repaired aortic neck, the inferior mesenteric artery (arrow), and the two renal arteries (*) that emerged from the aneurysm sac (the renal isthmus has already been sectioned).

The patient underwent open surgical treatment, with a transperitoneal approach, via median xypho-pubic laparotomy. Intraoperatively, after a longitudinal incision to open the retroperitoneal space, a large (intact) abdominal aneurysm was found emerging below the right renal artery, immediately below the inferior mesenteric artery (which had a high origin), with the left renal artery emerging from the aneurysm sac and a second renal artery emerging just below the primary left renal artery (Figure 3).

Initially, the proximal aneurysm neck was dissected and repaired (below the right renal artery and left superior polar artery), followed by dissection and repair of the inferior mesenteric artery and the two renal arteries that originated from the AAA (Figure 3), followed by dissection and repair of the internal and external iliac arteries bilaterally (Figure 44B). The horseshoe kidney was identified, fused at the inferior poles by a narrow longitudinal band, apparently fibrotic, as had been seen on preoperative tomography. Next, the renal isthmus was carefully dissected and sectioned longitudinally to achieve adequate exposure of the aorta, followed by renal hemostasis using teflon patch reinforced “U” sutures (Figures 55B). Immediately cranial to the renal isthmus, there were a small-caliber arterial branch and a small-caliber venous branch that crossed anterior of the AAA. These vessels had been identified in advance on preoperative angiotomography as responsible for arterial supply and venous drainage of the right inferior renal pole and were ligated and transected to obtain adequate exposure to the AAA.

**Figure 4:**
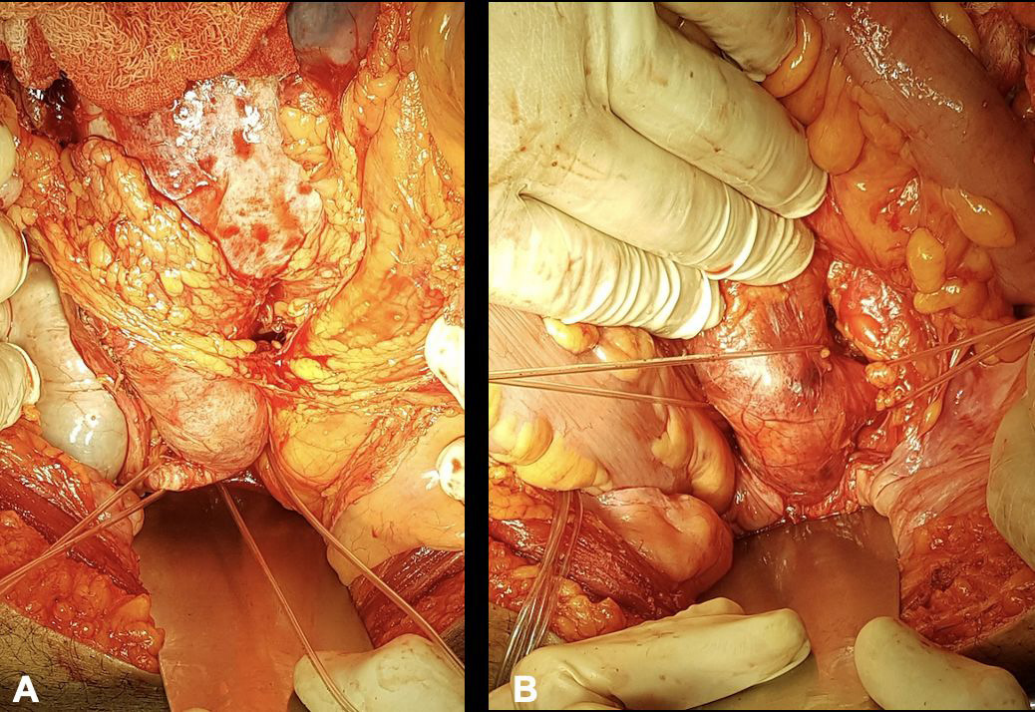
Surgical exposure of the right iliac bifurcation (**A**) and the left iliac bifurcation (**B**).

**Figure 5:**
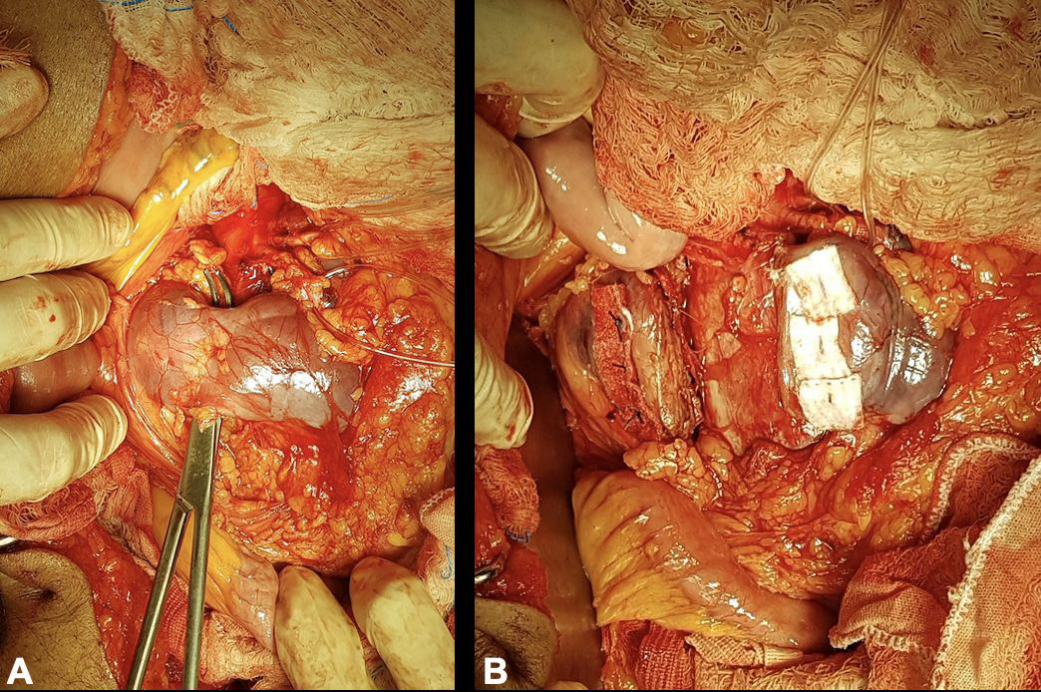
Surgical exposure of the renal isthmus before transection (**A**) and after transection (**B**).

**Figure 6:**
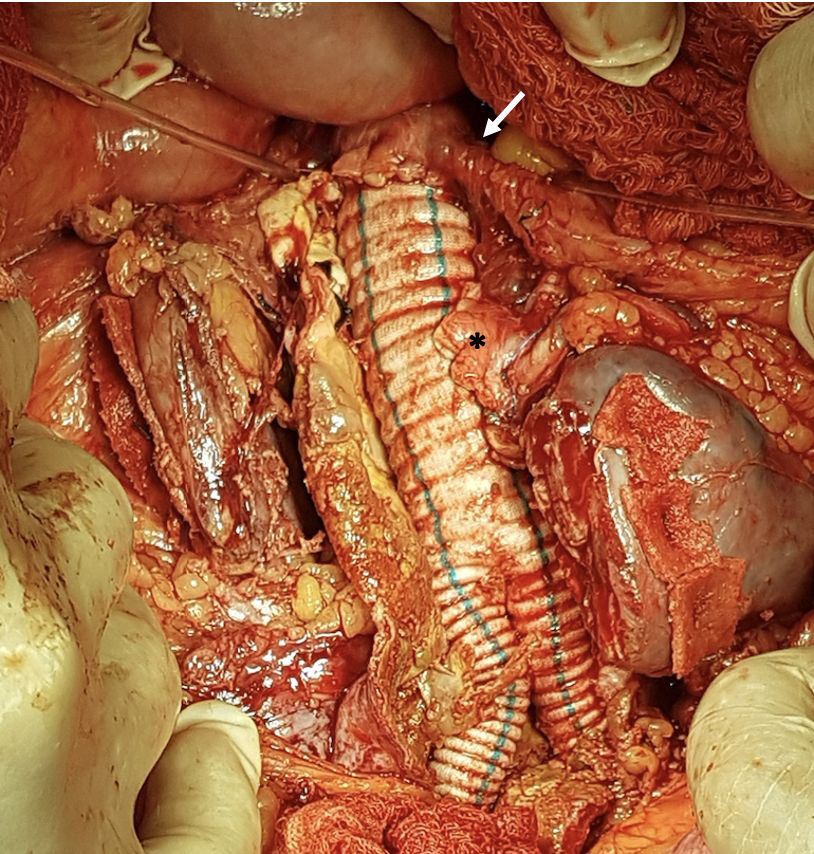
Surgical exposure revealing the proximal aortic anastomosis to the 18x9 mm Dacron graft, immediately below the origin of the inferior mesenteric artery (arrow) and reimplantation by patch of the two anomalous renal arteries onto the main body of the Dacron graft (*).

After systemic heparinization, with control of activated coagulation time, and arterial clamping, the aneurysm sac was opened longitudinally. Hemostasis of the patent lumbar arteries was achieved, followed by proximal anastomosis of an 18x9 mm Dacron graft to the infra-renal aorta, immediately below the origin of the inferior mesenteric artery. Next, the two renal arteries that emerged from the AAA were reimplanted, in a single patch, to the main body of the Dacron graft (Figure 6). At this point, the aortic clamp was moved to the distal portion of the main body of the Dacron graft. The two renal arteries that emerged from the AAA were clamped for 30 minutes. Next, the right branch of the Dacron graft was anastomosed to the right iliac bifurcation and the left branch was anastomosed to the left external iliac artery. The left internal iliac artery was ligated.

**Figure 7:**
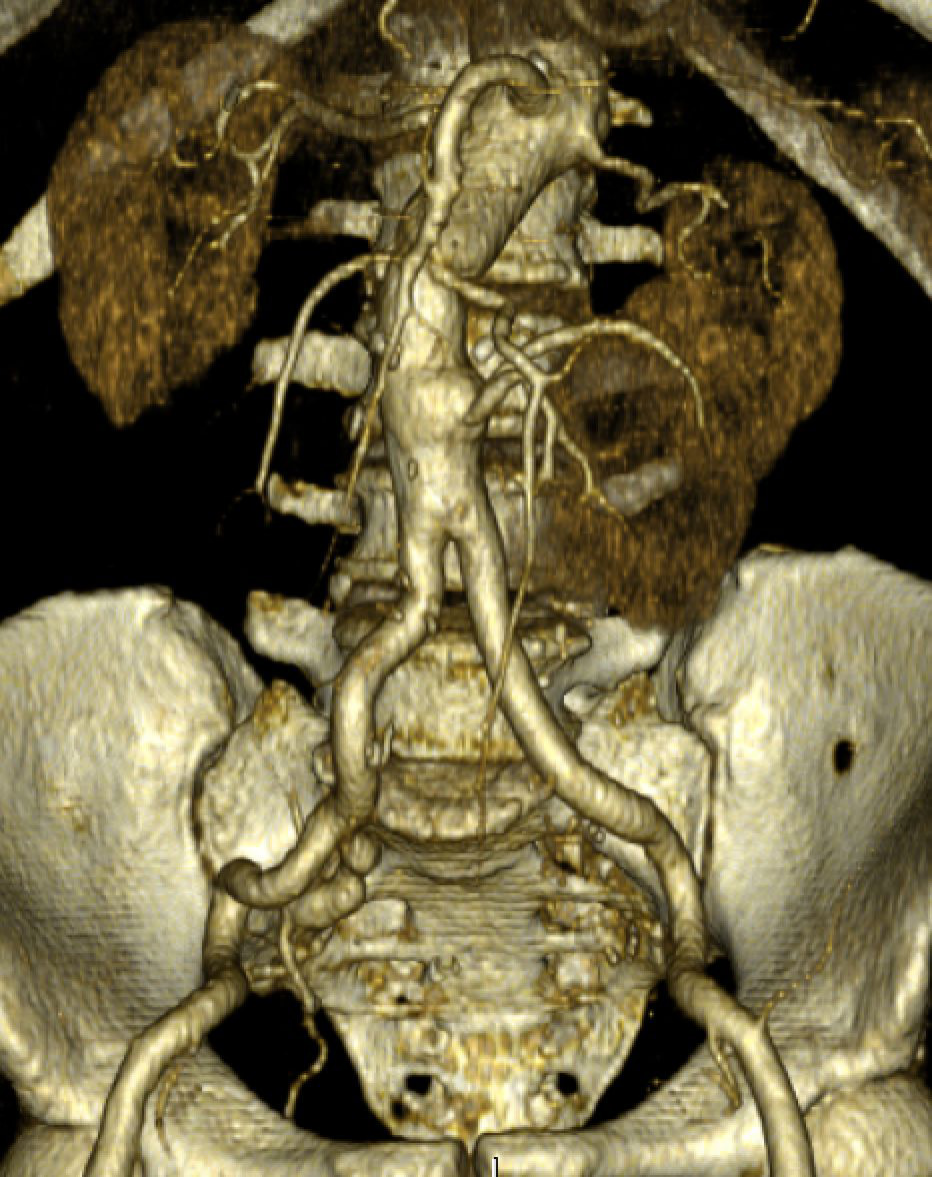
Postoperative angiotomography showing patent arterial anastomoses, patent reimplanted renal arteries, and good contrast uptake in the renal parenchyma bilaterally, with the exception of the right lower renal pole.

The patient was transferred to the intensive care unit for the immediate postoperative period, where he remained for 3 days, with an uneventful recovery and maintaining normal creatinine levels while in hospital. He was discharged home on the 8th postoperative day. Before hospital discharge, he underwent a control angiotomography, which showed that the arterial anastomoses and the reimplanted anomalous renal arteries were all patent. The images showed good contrast uptake in the renal parenchyma bilaterally, with the exception of an absence of contrast in the right inferior renal pole (Figure 7). At a late outpatient follow-up visit, on the 30th postoperative day, the patient was free from complaints and had normal serum creatinine levels.

## DISCUSSION

Several different surgical approaches have been used for AAA repair in patients with a horseshoe kidney. The transperitoneal approach offers better exposure of the aneurysm and the kidney, but the renal isthmus hinders adequate exposure of the AAA. The retroperitoneal approach offers the advantage of avoiding manipulation of the renal isthmus and the urinary tract, but there is limited access to the distal right common iliac artery (and its bifurcation).^4^ Regarding endovascular treatment, one advantage is that it avoids the difficulty of surgical exposure of the aorta caused by the horseshoe kidney that covers it.^5^ However, endovascular treatment is associated with a greater risk of postoperative renal infarction, since vascularization of horseshoe kidneys is highly variable and accessory renal arteries that originate in the AAA are usually responsible for a non-negligible proportion of the renal parenchyma. As has been demonstrated previously,^6^ each of these accessory renal arteries supplies a specific area of the renal parenchyma, with no collateral circulation between them. Thus, exclusion of these renal arteries with anomalous origins with an aortic endograft could cause renal infarcts and consequent loss of renal function.^7^

In the present case, at least two thirds of the left kidney and the inferior pole of the right kidney were supplied by the two renal arteries that originated in the AAA, each of which had a diameter of 3.5 mm, making an attempt at endovascular repair very difficult. Another important factor is that the surgery was performed in an urgent scenario, because of the patient’s acute symptoms.

Whether or not to preserve accessory renal arteries is another technical decision that must be taken when performing open repair. The majority of authors recommend preserving renal arteries with significant diameters (usually > 2 mm), since ligating them could cause renal ischemia and infarction during the postoperative period, with a negative impact on renal function.^8^ The accessory renal arteries can be reimplanted directly onto the graft body or by construction of a Carrel patch, which was our option because of the proximity of their aortic origins. They were therefore reimplanted using a single patch (Figure 6).

The renal isthmus may be composed of fibrotic tissue or of well-vascularized parenchymatous tissue, which is the most common scenario.^9^ In the few cases in which the isthmus is composed of fibrotic, non-functional tissue, sectioning it is described as the best option if a transperitoneal approach has been chosen,^10^ because preserving it makes adequate exposure of the AAA difficult.^11^ In the present case, the renal fusion was by a narrow fibrotic band connecting the inferior poles and so the decision was taken to transect it. In cases in which there is functioning parenchyma in the isthmus, sectioning it should be avoided, because, in addition to the greater technical difficulty, there is also a greater risk of contamination of the field and subsequent graft infection, since some horseshoe kidneys have chronic infection.^10^

We conclude that, in the present case, considering the anatomy of arterial supply and the fibrotic nature of the isthmus of the horseshoe kidney identified in advance on the preoperative angiotomography, in addition to the patient’s acute symptoms, open transperitoneal surgical treatment was a good therapeutic option.
