# Supplementary material for: Symptomatic abdominal aortic aneurysm in a patient with a horseshoe kidney
Source: J Vasc Bras. 2020 Dec 11;19:e20200088. doi: 10.1590/1677-5449.200088 (PMC8218002; doi:10.1590/1677-5449.200088)
Supplement: Translation

# Translation: Aneurisma de aorta abdominal sintomático associado a rim em ferradura

**Authors:** Geciana Maria Araujo Coelho, Ranielli Auxiliadora Assem França, Renan Danilo Lima da Rocha, Mariana de Oliveira Pantoja, Patricia de Souza Lacerda, Jose Emerson dos Santos Souza, Marcos Velludo Bernardes, Leonardo Pessoa Cavalcante

**Affiliations:** 1 Universidade Federal do Amazonas, Hospital Universitário Francisca Mendes, Serviço de Cirurgia Vascular e Endovascular, Manaus, AM, Brasil.; 2 Universidade Federal do Amazonas – UFAM, Hospital Universitário Getúlio Vargas, Serviço de Cirurgia Vascular, Manaus, AM, Brasil.; 3 Fundação Hospital Adriano Jorge – HUGV, Serviço de Cirurgia Geral, Manaus, AM, Brasil.; 4 Universidade do Estado do Amazonas – UEA, Escola Superior de Ciências da Saúde, Departamento de Clínica Cirúrgica, Manaus, AM, Brasil.; 5 Universidade Federal do Amazonas – UFAM, Faculdade de Medicina, Programa de Pós-graduação em Cirurgia - PPGRACI, Manaus, AM, Brasil.; 6 Universidade Federal do Amazonas – UFAM, Faculdade de Medicina, Departamento de Clínica Cirúrgica, Manaus, AM, Brasil.

**Keywords:** anormalidades congênitas, aneurisma aórtico, aneurisma da aorta abdominal, rim fundido, rim/anormalidades

## Abstract

O rim em ferradura é a anomalia congênita renal mais comum, ocorrendo em 0,15 a 0,25% de nascidos vivos. A associação de rim em ferradura com aneurisma de aorta abdominal é rara, estando presente em apenas 0,12% dos pacientes submetidos a tratamento dos aneurismas de aorta abdominal. Este desafio terapêutico consiste em um caso de um paciente portador de um aneurisma de aorta abdominal sintomático associado a rim em ferradura. A irrigação do rim em ferradura dava-se por meio de quatro artérias, sendo que duas delas emergiam diretamente do saco aneurismático. O caso foi solucionado por meio de cirurgia aberta, em caráter de urgência, com acesso transperitoneal, interposição de enxerto de Dacron bifurcado aorto bi-ilíaco e reimplante das duas artérias renais anômalas no corpo principal do Dacron. O paciente teve boa evolução pós-operatória, recebendo alta da unidade de tratamento intensivo no 3º dia e alta hospitalar no 8º dia, mantendo níveis normais de creatinina sérica.

## INTRODUÇÃO

O rim em ferradura é a anomalia congênita renal mais comum, ocorrendo em 0,15 a 0,25% de nascidos vivos, com predominância no sexo masculino na razão de 2:1^1^. A associação de rim em ferradura com aneurisma de aorta abdominal (AAA) é rara, estando presente em apenas 0,12% dos pacientes submetidos à correção cirúrgica, aberta ou endovascular, dos AAA^2^. O tratamento de um AAA associado a um rim em ferradura envolve algumas particularidades: 1) a presença do parênquima renal recobrindo o AAA; 2) as anomalias na irrigação arterial renal; e 3) as anomalias associadas do sistema coletor urinário^3^.

## PARTE I: SITUAÇÃO CLÍNICA

Um paciente do sexo masculino, 65 anos, tabagista, apresentando um AAA com 7,2 cm de diâmetro, assintomático, vinha em acompanhamento ambulatorial, realizando exames pré-operatórios para estratificação do risco cirúrgico. Durante o período de acompanhamento ambulatorial, o paciente apresentou dor abdominal aguda e de forte intensidade, irradiando para a região lombar bilateralmente, sem hipotensão associada. Foi, então, internado em caráter de urgência.

Na angiotomografia pré-operatória, observava-se a presença de um rim em ferradura, no qual a fusão dava-se pelos polos renais inferiores, com componente fibrótico estreito na zona de fusão, anteriormente ao AAA (Figura 1). O rim direito era irrigado, majoritariamente, por uma artéria renal principal, que emergia poucos milímetros abaixo da artéria mesentérica superior; o rim esquerdo era irrigado por uma artéria polar superior esquerda, que emergia, também, poucos milímetros abaixo da artéria mesentérica superior e por uma artéria renal principal (hilar), que emergia do segmento proximal do AAA e media 3,5 mm de diâmetro. Os polos renais inferiores eram irrigados por uma segunda artéria (acessória), que também se originava do segmento proximal do AAA, aproximadamente 3 mm abaixo da artéria renal esquerda principal, medindo também 3,5 mm de diâmetro. A artéria renal esquerda acessória se bifurcava em dois ramos: um deles irrigava o polo renal inferior esquerdo e o outro, que cruzava anteriormente o AAA, acima do istmo do rim em ferradura, irrigava o polo renal inferior direito (Figura 2). A artéria mesentérica inferior tinha origem alta, no segmento distal do colo proximal do AAA, media 6 mm de diâmetro e estava pérvia. O AAA iniciava-se 25 mm abaixo da artéria renal direita, com colo apresentando angulação anterior de aproximadamente 60 graus, e acometia as duas artérias ilíacas comuns (29 mm de dilatação no lado direito e 39 mm de dilatação no lado esquerdo).

**Figura 1:** Rim em ferradura com fusão anterior ao aneurisma de aorta abdominal.

**Figura 2:** Desenho esquemático evidenciando irrigação do rim em ferradura e origem das artérias digestivas. *: Artéria mesentérica inferior com origem alta.

Diante dessa situação, havia algumas possibilidades terapêuticas: 1) tratamento endovascular; 2) tratamento cirúrgico aberto com acesso transperitoneal; ou 3) tratamento cirúrgico aberto com acesso retroperitoneal. O paciente consentiu com a publicação de seu caso.

## PARTE II: O QUE FOI FEITO

O paciente foi submetido a tratamento cirúrgico aberto, transperitoneal, por meio de laparotomia mediana xifo-púbica. No intraoperatório, após a abertura longitudinal do retroperitônio, foi evidenciado volumoso aneurisma abdominal (íntegro) iniciando-se abaixo da artéria renal direita, imediatamente abaixo da artéria mesentérica inferior (emergência alta da mesma), com artéria renal esquerda emergindo do aneurisma e uma segunda artéria renal emergindo imediatamente abaixo da renal esquerda principal (Figura 3).

**Figura 3:** Exposição cirúrgica do colo aórtico reparado, bem como da artéria mesentérica inferior (seta) e das duas artérias renais (*) que emergiam do saco aneurismático (istmo renal já seccionado).

Inicialmente, foram realizados dissecção e reparo do colo proximal do aneurisma (abaixo da artéria renal direita e da polar superior esquerda), dissecção e reparo da mesentérica inferior, bem como das duas artérias renais que se originavam do AAA (Figura 3), seguidos de dissecção e reparo das artérias ilíacas internas e externas bilateralmente (Figura 44B). Foi identificado o rim em ferradura, unido pelos polos inferiores por meio de uma faixa longitudinal estreita, aparentemente fibrótica, conforme evidenciado na tomografia pré-operatória. Em seguida, foram realizadas dissecção cuidadosa e secção longitudinal do istmo renal para uma exposição aórtica adequada, seguidas de hemostasia renal com pontos em “U” ancorados com barra de teflon (Figura 55B). Imediatamente cranial ao istmo renal, havia um ramo arterial e um ramo venoso que cruzavam anteriormente o AAA, de calibres finos, identificados previamente na angiotomografia pré-operatória como responsáveis pela irrigação arterial/drenagem venosa do polo renal inferior direito, tendo sido ligados e seccionados para obtenção de acesso adequado ao AAA.

**Figura 4:** Exposição cirúrgica da bifurcação ilíaca direita (**A**) e esquerda (**B**).

**Figura 5:** Exposição cirúrgica do istmo renal pré-secção (**A**) e pós-secção (**B**).

Após heparinização sistêmica, com controle do tempo de coagulação ativada, e clampeamento arterial, o aneurisma foi aberto longitudinalmente. Foi realizada a hemostasia das artérias lombares pérvias, e procedeu-se a anastomose proximal da prótese de Dacron 18x9 mm na aorta infra-renal, imediatamente abaixo da emergência artéria mesentérica inferior. Em seguida, as duas artérias renais que emergiam do AAA foram reimplantadas, em *patch* único, no corpo principal da prótese de Dacron (Figura 6). Nesse momento, o clampe aórtico foi movido para o segmento distal do corpo principal da prótese de Dacron, sendo de 30 minutos o tempo de clampeamento das duas artérias renais que emergiam do AAA. Em seguida, o ramo direito da prótese de Dacron foi anastomosado à bifurcação ilíaca direita e o ramo esquerdo anastomosado à artéria ilíaca externa esquerda, tendo a artéria ilíaca interna esquerda sido ligada.

**Figura 6:** Exposição cirúrgica evidenciando a anastomose aórtica proximal da prótese de Dacron 18x9 mm, imediatamente abaixo da emergência da artéria mesentérica inferior (seta) e o reimplante do *patch* das duas artérias renais anômalas no corpo principal do Dacron (*).

O paciente foi encaminhado à unidade de terapia intensiva no pós-operatório imediato, onde permaneceu por 3 dias, evoluindo sem intercorrências e mantendo valores normais de creatinina durante a internação. Ele recebeu alta hospitalar no 8º dia pós-operatório. Antes da alta hospitalar, o paciente foi submetido a angiotomografia de controle, que evidenciou perviedade das anastomoses arteriais, bem como das artérias renais anômalas reimplantadas. O exame apresentou boa contrastação do parênquima renal bilateralmente, exceto pela ausência de contrastação do polo renal inferior direito (Figura 7). No retorno ambulatorial tardio, no 30º dia pós-operatório, o paciente encontrava-se sem queixas e mantinha níveis séricos normais de creatinina.

**Figura 7:** Angiotomografia pós-operatória evidenciando perviedade das anastomoses arteriais, bem como das artérias renais reimplantadas e boa contrastação do parênquima renal bilateralmente, exceto do polo renal inferior direito.

## DISCUSSÃO

Várias abordagens cirúrgicas têm sido usadas para o reparo de AAA em pacientes com rim em ferradura. A abordagem transperitoneal proporciona a melhor exposição do aneurisma e do rim, porém a presença do istmo renal prejudica a exposição adequada do AAA. A abordagem retroperitoneal tem a vantagem de evitar a manipulação do istmo e do trato urinário; no entanto, o acesso à artéria ilíaca comum direita distal (e de sua bifurcação) torna-se limitado^4^. Quanto ao tratamento endovascular, há a vantagem de evitar-se a dificuldade da exposição cirúrgica da aorta em decorrência da presença do rim em ferradura que a recobre^5^. Todavia, no tratamento endovascular há maior risco de infartos renais pós-operatórios, visto que a vascularização do rim em ferradura é muito variável e, por vezes, artérias renais acessórias originadas do AAA são responsáveis pela irrigação de quantidade não desprezível de parênquima renal. Como já demonstrado^6^, cada uma dessas artérias renais acessórias irriga uma área específica de parênquima renal, não havendo circulação colateral entre elas. Desse modo, a exclusão dessas artérias renais de origem anômala com a endoprótese aórtica pode levar a infartos renais, com consequente perda de função renal^7^.

No presente caso, pelo menos dois terços do rim esquerdo e o polo inferior do rim direito eram irrigados pelas duas artérias renais que se originavam do AAA, com 3,5 mm de diâmetro cada, o que dificultaria sobremaneira o reparo endovascular. Outro fator importante é que a cirurgia ocorreu em um cenário de urgência, dada a sintomatologia aguda do paciente.

A preservação ou não das artérias acessórias renais é outra decisão técnica a ser tomada durante o reparo aberto. A maioria dos autores recomendam preservar artérias renais com diâmetro significativo (usualmente > 2 mm), visto que a ligadura delas pode acarretar isquemia e infarto renal no pós-operatório, com impacto negativo na função renal^8^. O reimplante das artérias renais acessórias pode ser realizado diretamente ao corpo da prótese ou através da confecção de *patch* de Carrel, tendo sido essa última opção a escolhida por nós em decorrência da proximidade na origem aórtica das duas. Assim, foram reimplantadas em um *patch* único (Figura 6).

Quanto ao istmo renal, ele pode ser constituído por tecido fibrótico ou por tecido parenquimatoso bem vascularizado, sendo o último o cenário mais frequente^9^. Nos poucos casos em que o istmo é constituído por tecido fibrótico e não funcionante, caso escolhido o acesso transperitoneal, descreve-se como melhor tática a sua secção^10^, visto que sua preservação dificulta a exposição adequada do AAA^11^. No presente caso, a fusão renal dava-se pelos polos inferiores e constituía-se por uma faixa estreita de tecido fibrótico, tendo sido optado por sua divisão. Nos casos em que há parênquima funcionante no istmo, deve-se evitar a secção dele, pois, além de maior dificuldade técnica, há também maior risco de contaminação do campo e subsequente infecção da prótese, visto que, em alguns rins em ferradura, há a presença de infecção crônica^10^.

Concluímos que, no presente caso, considerando a anatomia da irrigação arterial e a caraterística fibrótica do istmo do rim em ferradura previamente identificada na angiotomografia pré-operatória, bem como a sintomatologia aguda apresentada pelo paciente, o tratamento cirúrgico aberto transperitoneal foi uma boa opção terapêutica.
